# Monocytic HLA-DR expression kinetics in septic shock patients with different pathogens, sites of infection and adverse outcomes

**DOI:** 10.1186/s13054-020-2830-x

**Published:** 2020-03-20

**Authors:** Guus P. Leijte, Thomas Rimmelé, Matthijs Kox, Niklas Bruse, Céline Monard, Morgane Gossez, Guillaume Monneret, Peter Pickkers, Fabienne Venet

**Affiliations:** 1 Department of Intensive Care Medicine, Radboud University Medical Center, Nijmegen, The Netherlands; 2 Radboud Center for Infectious Diseases, Radboud University Medical Center, Nijmegen, The Netherlands; 3 Pathophysiology of Injury-Induced Immunosuppression, Université Claude Bernard Lyon 1, Hospices Civils de Lyon, bioMérieux, Edouard Herriot Hospital, 5 place d’Arsonval, 69437 Lyon Cedex 03, France; 4 Anesthesia and Critical Care Medicine Department, Hospices Civils de Lyon, Edouard Herriot Hospital, Lyon, France; 5 Immunology Laboratory, Hospices Civils de Lyon, Edouard Herriot Hospital, Lyon, France

**Keywords:** mHLA-DR, Septic shock, Trajectory analysis, Site of infection, Pathogens, Mortality, Secondary infections, Adverse outcome, Infection-free survival

## Abstract

**Background:**

Decreased monocytic (m)HLA-DR expression is the most studied biomarker of sepsis-induced immunosuppression. To date, little is known about the relationship between sepsis characteristics, such as the site of infection, causative pathogen, or severity of disease, and mHLA-DR expression kinetics.

**Methods:**

We evaluated mHLA-DR expression kinetics in 241 septic shock patients with different primary sites of infection and pathogens. Furthermore, we used unsupervised clustering analysis to identify mHLA-DR trajectories and evaluated their association with outcome parameters.

**Results:**

No differences in mHLA-DR expression kinetics were found between groups of patients with different sites of infection (abdominal vs. respiratory, *p* = 0.13; abdominal vs. urinary tract, *p* = 0.53) and between pathogen categories (Gram-positive vs. Gram-negative, *p* = 0.54; Gram-positive vs. negative cultures, *p* = 0.84). The mHLA-DR expression kinetics differed between survivors and non-survivors (*p* < 0.001), with an increase over time in survivors only. Furthermore, we identified three mHLA-DR trajectories (‘early improvers’, ‘delayed or non-improvers’ and ‘decliners’). The probability for adverse outcome (secondary infection or death) was higher in the delayed or non-improvers and decliners vs. the early improvers (delayed or non-improvers log-rank *p* = 0.03, adjusted hazard ratio 2.0 [95% CI 1.0–4.0], *p* = 0.057 and decliners log-rank *p* = 0.01, adjusted hazard ratio 2.8 [95% CI 1.1–7.1], *p* = 0.03).

**Conclusion:**

Sites of primary infection or causative pathogens are not associated with mHLA-DR expression kinetics in septic shock patients. However, patients showing delayed or no improvement in or a declining mHLA-DR expression have a higher risk for adverse outcome compared with patients exhibiting a swift increase in mHLA-DR expression. Our study signifies that changes in mHLA-DR expression over time, and not absolute values or static measurements, are of clinical importance in septic shock patients.

## Background

Sepsis is defined as a life-threatening organ dysfunction due to a dysregulated host response to infection [1]. Immunological heterogeneity is a well-known phenomenon in sepsis patients, as they may present with both hyperinflammatory and immunosuppressive phenotypes. This might explain the failure of one-fits-all approaches and illustrates that a personalized approach is required for this complex syndrome [2]. Biomarkers to assess the individual immune status of a patient are currently not used in clinical practice but are essential to facilitate precision medicine in sepsis patients. Decreased monocytic (m)HLA-DR expression is the most studied biomarker of sepsis-induced immune suppression [3–5]. Multiple studies established associations between mHLA-DR expression and outcome parameters, such as secondary infections and mortality [6–10]. Moreover, there are indications from small studies that mHLA-DR expression differs between patients with different causative pathogens and/or sites of infection [11, 12]. However, little is known about mHLA-DR expression kinetics and their relationship with sepsis characteristics and severity of disease. Detailed insight in mHLA-DR expression kinetics between subgroups of septic patients could pave the way towards increased understanding of sepsis immunopathology and aid the selection of patients that could benefit from immunostimulatory agents. In the current study, we set out to evaluate whether the kinetics of mHLA-DR expression, measured using a standardized assay, vary between patients with different primary sites of infection and different pathogens in a large cohort of septic shock patients. Furthermore, using unsupervised clustering analysis, we aimed to identify specific mHLA-DR trajectories and relate these to outcome parameters such as the occurrence of secondary infections and mortality.

## Methods

### Patients and data collection

Between April 2014 and June 2018, all patients at the intensive care unit (ICU) of Edouard Herriot Hospital (Hospices Civils de Lyon, France) were screened daily for septic shock by a dedicated research team. Before 2016, the diagnosis of septic shock was based on the ‘old’ criteria, and after 2016, patients were enrolled based on the Sepsis-3 criteria [1]. Patients were treated according to the Surviving Sepsis Campaign recommendations and received intravenous antimicrobials as soon as possible and source control if required. Intravenous hydrocortisone (200 mg daily) was administered in case of refractory septic shock. After meeting the inclusion criteria and when non-opposition was acquired, blood was withdrawn and data were collected. The exclusion criteria and data collection methods are detailed in the Supplementary Material. In order to increase homogeneity and generalizability of our cohort and to comply with the current sepsis definition, we retrospectively applied the Sepsis-3 criteria of septic shock (sepsis requiring vasopressor therapy and lactate ≥ 2 mmol/L) on patients included before 2016. Patients who did not meet the Sepsis-3 septic shock criteria were excluded from further analyses. The local ethical board (#IRB11236) waived the need for written informed consent for this observational study, which is registered at the French Ministry of Research and Teaching (#DC-2008-509) and at Clinicaltrials.gov (NCT02803346).

### Analysis of HLA-DR expression on monocytes

mHLA-DR expression, in numbers of antibodies bound per monocyte (AB/cell), was analysed at three time points (day 1 or 2, day 3 or 4 and day 6, 7 or 8) using the Anti-HLA-DR/Anti-Monocyte Quantibrite assay (BD Biosciences, San Jose, USA), as detailed in the Supplementary Material.

### Statistical analysis

A flowchart describing the patient datasets used for the different analyses is provided in Supplementary Fig. 1. Distribution of data, assessed using Shapiro-Wilk tests, was non-parametrical and therefore presented as median [interquartile range]. Categorical data were tested using chi-squared tests and continuous data were tested using Student’s *t* test on log-transformed data. Within-group differences over time were assessed using (repeated measures) one-way analysis of variance (ANOVA) on log-transformed data with Dunnett’s post hoc test, whereas between-groups differences over time were tested using repeated measures two-way ANOVA (time*group interaction term) on log-transformed data. For the analysis of secondary infection over time, mHLA-DR data was censored when a secondary infection occurred. Pearson’s correlation as well as linear and Cox proportional-hazards regression analysis were performed after log-transformation of continuous data. Unsupervised cluster analysis was performed using a software (*crimCV* package in R) to fit a finite mixture of zero-inflated Poisson (ZIP) models on our longitudinal data in order to calculate the group-based trajectories [13]. The model selection and bootstrap procedure is described in the Supplementary Material and optimal model performance was attained using a 2nd-degree polynomial fit and 4 clusters. A *p* value less than 0.05 was considered statistically significant. Data were analysed using SPSS Statistics version 22 (IBM Corporation, Armonk, NY, USA), Graphpad Prism version 5.03 (Graphpad Software, La Jolla, USA) and R version 3.5.3.

## Results

### Patient characteristics

We included 241 septic shock patients of which the characteristics are listed in Table 1. mHLA-DR was determined in 203 patients on days 1–2 (mHLA-DR expression of 4709 AB/cell [2984–7386]), 206 patients on days 3–4 (4838 AB/cell [2972–7715]), and 133 patients on days 6–8 (6507 AB/cell [4104–9353]), yielding a significantly increased expression over time (*p* = 0.02). Circulating monocyte counts did not significantly differ between the three time points (data not shown).

**Table 1:** Clinical characteristics of septic shock patients within the total cohort and for different sites of infection and pathogens

	Total cohort (***n*** = 241)	Abdominal infection (***n*** = 107)	Respiratory infection (***n*** = 44)	Urinary tract infection (***n*** = 29)	***p*** value	Gram-positive bacteria (***n*** = 64)	Gram-negative bacteria (*n* = 77)	Negative cultures (***n*** = 65)	***p*** value
**Baseline**									
Gender (male)	158 (66%)	66 (62%)	31 (71%)	15 (52%)	0.27	43 (67%)	46 (60%)	47 (72%)	0.28
Age (years)	70 [62–78]	69 [60–78]	71 [64–80]	74 [67–81]	0.38	66 [56–77]	71 [64–80]	71 [64–76]	0.05
BMI	27 [22–31]	26 [23–32]	24 [21–27]	28 [24–37]	0.005	26 [21–31]	27 [23–31]	25 [22–30]	0.46
SOFA score (< 24 h)	9 [8–12]	8 [8–11]	12 [10–14]	10 [8–12]	0.001	9 [6–12]	9 [8–12]	10 [8–12]	0.15
SAPS II score (< 24 h)	62 [50–76]	61 [53–76]	73 [62–87]	59 [50–74]	0.02	56 [44–71]	61 [50–78]	65 [52–76]	0.29
Lactate (mmol/L)	3.5 [2.6–5.7]	3.9 [2.6–5.5]	3.8 [2.7–6.8]	3.7 [2.8–6.9]	0.79	3.4 [2.6–4.7]	4.0 [3.0–6.6]	3.3 [2.5–5.8]	0.22
**Interventions**									
Noradrenaline (μg/kg/min)^#^	0.8 [0.4–1.4]	0.8 [0.4–1.5]	1.0 [0.6–2.0]	0.8 [0.4–1.3]	0.14	0.8 [0.4–1.2]	0.7 [0.4–1.5]	0.8 [0.5–1.7]	0.16
Hydrocortisone	129 (54%)	57 (53%)	29 (66%)	13 (45%)	0.18	33 (52%)	41 (53%)	40 (62%)	0.47
Mechanical ventilation	195 (81%)	90 (84%)	39 (91%)	19 (66%)	0.02	49 (77%)	57 (74%)	54 (84%)	0.32
Length of MV (days)	3 [0.025–7]	3 [1–7]	4 [1–10]	1 [0–3]	0.39	3 [0–8]	3 [0–7]	2 [1–6]	0.18
Renal replacement therapy	39 (16%)	13 (12%)	4 (9%)	4 (14%)	0.80	15 (23%)	11 (14%)	10 (15%)	0.31
Post-surgery admission	143 (59%)	89 (83%)	5 (12%)	7 (24%)	< 0.0001	44 (69%)	36 (47%)	38 (59%)	0.04
**Outcome measures**									
Secondary infection	32 (13%)	16 (15%)	6 (14%)	0 (0%)	0.09	10 (16%)	10 (13%)	6 (9%)	0.55
Length of ICU stay (days)	7 [4–13]	8 [5–15]	7 [3–13]	4 [3–8]	0.002	7 [4–17]	8 [4–12]	6 [3–11]	0.16
Length of hospital stay (days)	21 [10–41]	22 [12–44]	13 [5–23]	17 [7–32]	0.04	29 [11–56]	22 [12–32]	14 [5–29]	0.002
28-day mortality	87 (36%)	32 (30%)	27 (61%)	6 (21%)	0.0002	23 (36%)	23 (30%)	31 (48%)	0.09
ICU mortality	77 (32%)	30 (28%)	25 (57%)	4 (14%)	0.0002	21 (33%)	22 (29%)	25 (39%)	0.46
Hospital mortality	124 (51%)	36 (37%)	27 (69%)	7 (27%)	0.001	24 (40%)	24 (35%)	32 (53%)	0.11

Data are presented as frequencies and percentages (%) for categorical data and medians and interquartile ranges [IQR] for continuous variables. *p* values were calculated using chi-squared tests for categorical data and one-way ANOVA on log-transformed continuous data

*BMI* body mass index, *SOFA* Sequential Organ Failure Assessment, *SAPS* Simplified Acute Physiology Score, *MV* mechanical ventilation, *ICU* intensive care unit

^#^Maximum dose within the first 24 h after study inclusion

### mHLA-DR expression kinetics in patients with different sites of infection

**Fig. 1:**
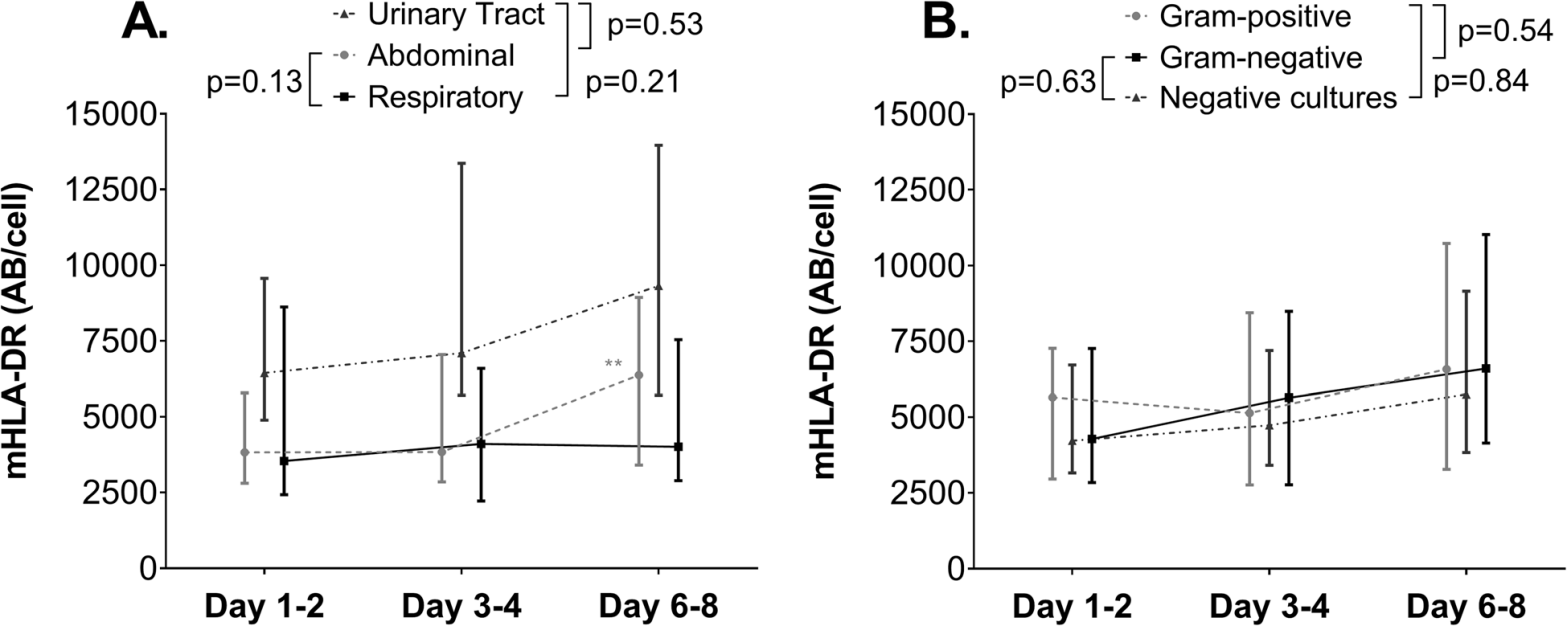
mHLA-DR expression in septic shock patients with **a** abdominal infections (*n* = 77), respiratory infections (*n* = 24) or urinary tract infections (*n* = 18), and **b** Gram-positive (*n* = 42) or Gram-negative bacteria (*n* = 55), or patients with negative cultures (*n* = 41). Data are presented as median [IQR] and *p* values were calculated using repeated measures two-way ANOVA on log-transformed data (time*group interaction term). **p < 0.01 within group over time calculated using repeated measures one-way ANOVA on log-transformed data

Patient characteristics specified according to the most prevalent sites of infection (abdominal, respiratory and urinary tract) are listed in Table 1. Patient numbers for all sites of infection are specified in Supplementary Table 1. Patients with a respiratory focus had a higher SOFA score at ICU admission and a higher mortality rate compared to patients with abdominal or urinary tract infections, whereas no differences were found between abdominal and urinary tract infections (Table 1). While the incidence of secondary infections did not differ between patients with a respiratory or abdominal focus, both groups had a higher incidence compared to the urinary tract group (Table 1). No differences in mHLA-DR expression were found between the respiratory and abdominal site of infection at any of the individual time points (Supplementary Fig. 2A). In accordance, no difference in mHLA-DR kinetics was observed between respiratory (*n* = 25) and abdominal (*n* = 77) sites of infection in the subgroups used for kinetics analysis (Fig. 1a). Nevertheless, mHLA-DR expression within the group of patients with an abdominal infection increased over time, whereas it remained unchanged within the respiratory focus group (Fig. 1a and Supplementary Fig. 2A). At days 1–2 and 3–4, patients with a urinary tract infection exhibited a higher mHLA-DR expression than patients with an abdominal focus (Supplementary Fig. 2A), whereas mHLA-DR expression in the urinary tract group was higher than in the respiratory infection group only at days 3–4 (Supplementary Fig. 2A). No between-group differences were found for urinary tract vs. abdominal or respiratory infection (Fig. 1a). The numbers of patients with other sites of infection were too low to draw conclusions about mHLA-DR expression kinetics.

### mHLA-DR expression kinetics in patients with different pathogens

Patient characteristics specified according to the most prevalent pathogen categories (patients with Gram-positive, Gram-negative and negative cultures) are listed in Table 1. Patient numbers for all pathogen categories are specified in Supplementary Table 2. SOFA score, ICU length of stay, incidence of secondary infections and 28-day mortality were not different across patients with Gram-positive, Gram-negative or negative cultures. No differences in mHLA-DR expression between the different pathogens categories were found in the total cohort at any of the individual time points (Supplementary Fig. 2B). Furthermore, no within-group differences over time were observed (Supplementary Fig. 2B). Accordingly, there were no within- or between-group differences in mHLA expression for patients with Gram-positive (*n* = 42), Gram-negative (*n* = 55) and negative cultures (*n* = 41) in the subgroups used for kinetics analysis (Fig. 1b). Fungi and viruses each represented 2% of the pathogens cultured. The mHLA-DR kinetics of patients with a fungal or viral infection did not reveal a distinct pattern when compared to bacterial sepsis (data not shown).

### Relationship between SOFA score, site of infection, type of pathogen and mHLA-DR expression

Across all patients, SOFA score at admission was inversely correlated with mHLA-DR expression at days 3–4 (*r* = − 0.22, *p* = 0.002) and 6–8 (*r* = − 0.30, *p* < 0.001). After adjustment for site of infection, causative pathogens and the use of hydrocortisone in a linear regression model, SOFA score at admission remained independently correlated with mHLA-DR expression at days 3–4 (*p* = 0.008) and day 6–8 (*p* = 0.002).

### Relationship between mHLA-DR expression and secondary infections

**Fig. 2:**
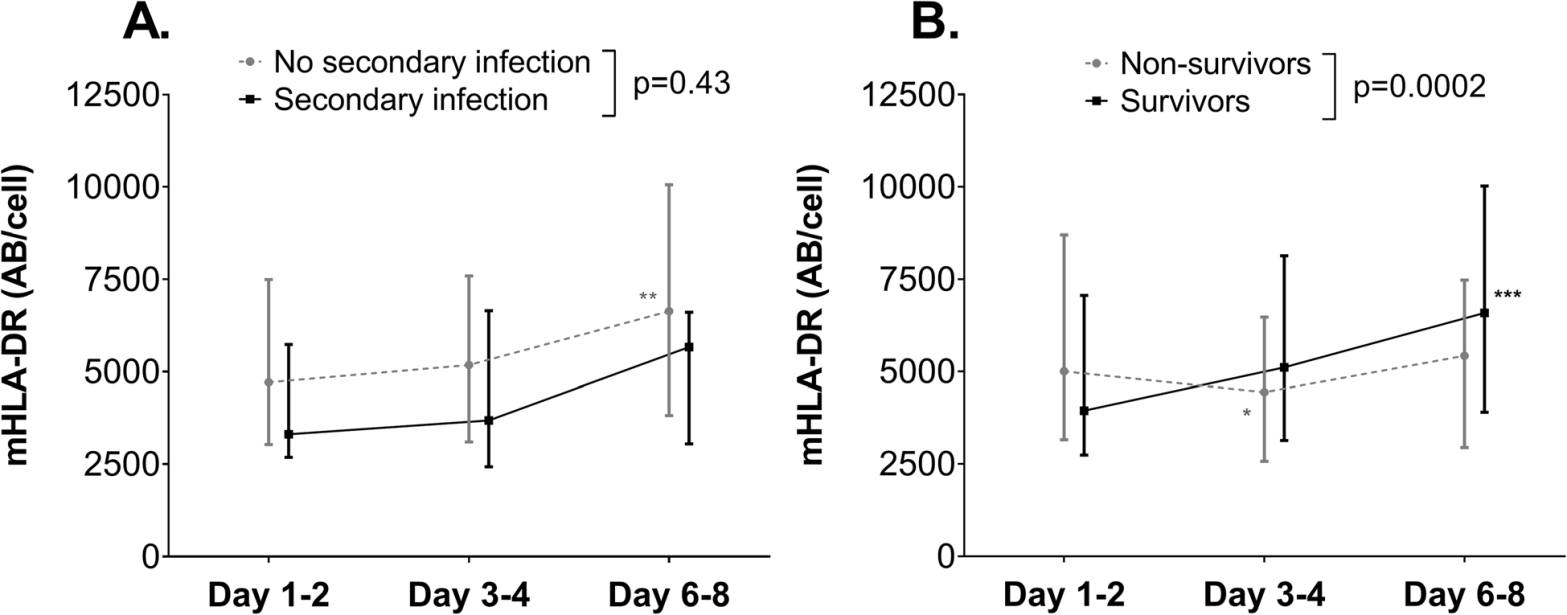
mHLA-DR expression of septic shock patients **a** who did or did not develop a secondary infection (*n* = 30 and *n* = 138, respectively) and **b** who did or did not survive until day 28 (*n* = 116 and *n* = 52, respectively). Data are presented as median [IQR] and *p* values were calculated using repeated measures two-way ANOVA on log-transformed data (time*group interaction term). *p < 0.05, **p < 0.01 and ***p < 0.001 within group over time calculated using repeated measures one-way ANOVA on log-transformed data

Thirteen percent of the patients developed a secondary infection, which occurred after 10 [7–20] days. The sites of secondary infections are listed in Supplementary Table 3. We did not find a difference in mHLA-DR kinetics between patients with (*n* = 30) or without (*n* = 138) secondary infection (Fig. 2a), although within-group analysis revealed a significant increase over time in patients who did not develop a secondary infection (Fig. 2a and Supplementary Fig. 2C). In line with earlier work of our group showing that mHLA-DR expression on days 3–4 correlated with secondary infections [6], we found a significantly lower mHLA-DR expression on days 3–4 in the patients who developed a secondary infection compared to patients who did not (*p* = 0.03, Supplementary Fig. 2C), whereas this difference was less pronounced on days 6–8 (*p* = 0.07). Therefore, we further explored the 3–4 days time point, but the AUROC of HLA-DR expression to predict secondary infections was poor (0.62 [95%CI 0.52–0.72]). Furthermore, the increase in mHLA-DR expression between days 3–4 and day 6–8 was not significantly different between patients with and without secondary infections (*p* = 0.17, data not shown).

### Relationship between mHLA-DR expression and mortality

The 28-day mortality in the total cohort was 36%. mHLA-DR expression kinetics in 28-day survivors (*n* = 116) were significantly different compared to non-survivors (*n* = 52, Fig. 2b). This was due to an overall increase in mHLA-DR expression kinetics in patients who survived, whereas the expression decreased from days 1–2 to days 3–4 in non-survivors (Fig. 2b). In contrast to earlier results of our group [7], mHLA-DR expression on days 3–4 was not significantly lower in non-survivors compared to survivors (Supplementary Fig. 2D), but expression on days 6–8 was (*p* = 0.001, Supplementary Fig. 2B). For mHLA-DR expression on days 6–8, the area under the receiver operator curve (AUROC) to predict mortality was poor (0.66 [95%CI 0.56–0.77]). The increase in mHLA-DR expression between days 3–4 and days 6–8 was significantly more pronounced in patients who were alive at day 28 compared to those who were not (*p* = 0.01 and AUROC, 0.65 [95%CI 0.54–0.77]).

### mHLA-DR trajectories and their relation with clinical outcome

**Fig. 3:**
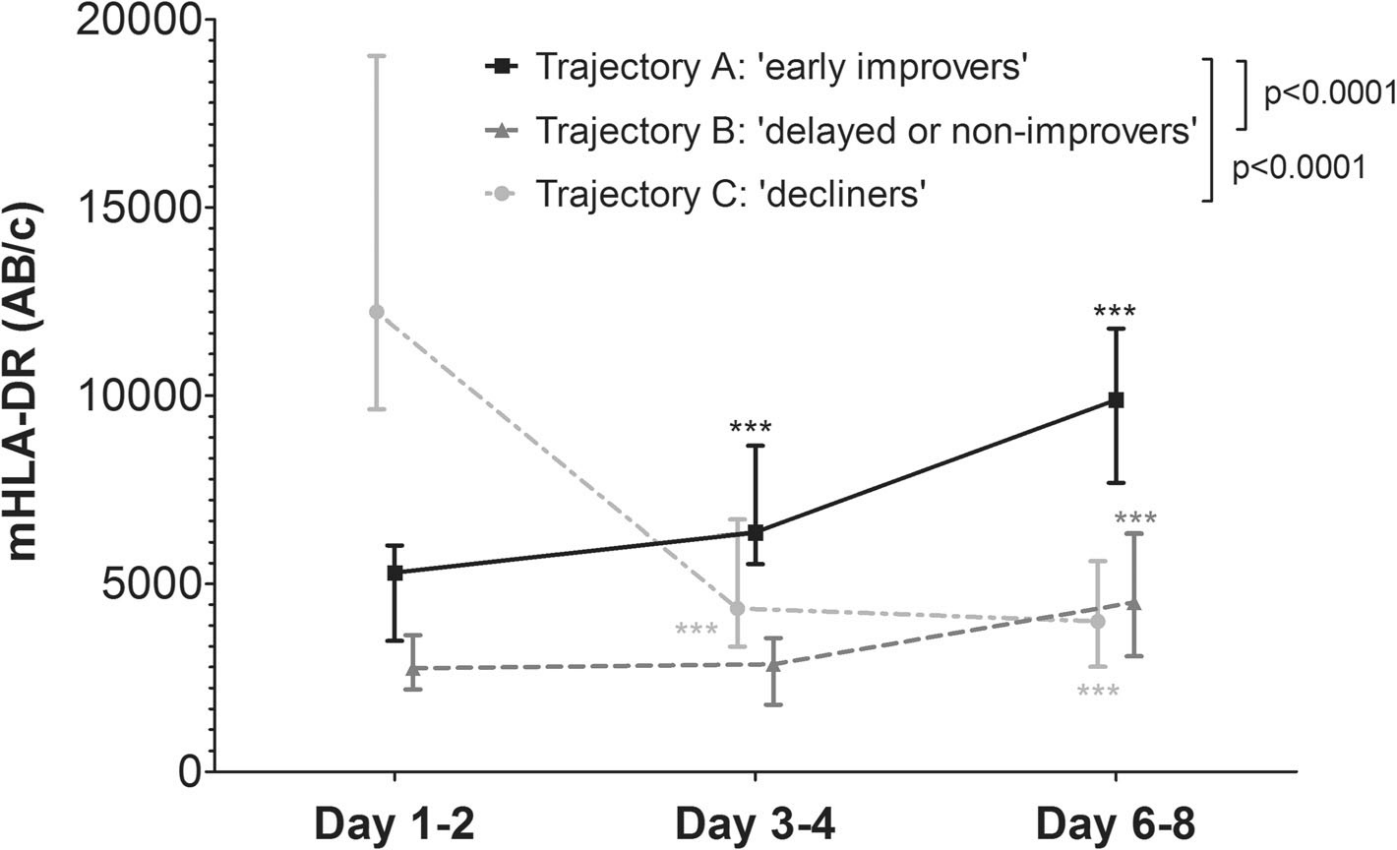
mHLA-DR trajectories identified by unsupervised clustering analysis of mHLA-DR expression kinetics. Data are presented as median [IQR] and *p* values were calculated using repeated measures two-way ANOVA on log-transformed data vs. reference trajectory A (time*group interaction term). ***p < 0.001 calculated with one-way ANOVA on log-transformed data with Dunnett’s multiple comparison test compared to baseline

**Fig. 4:**
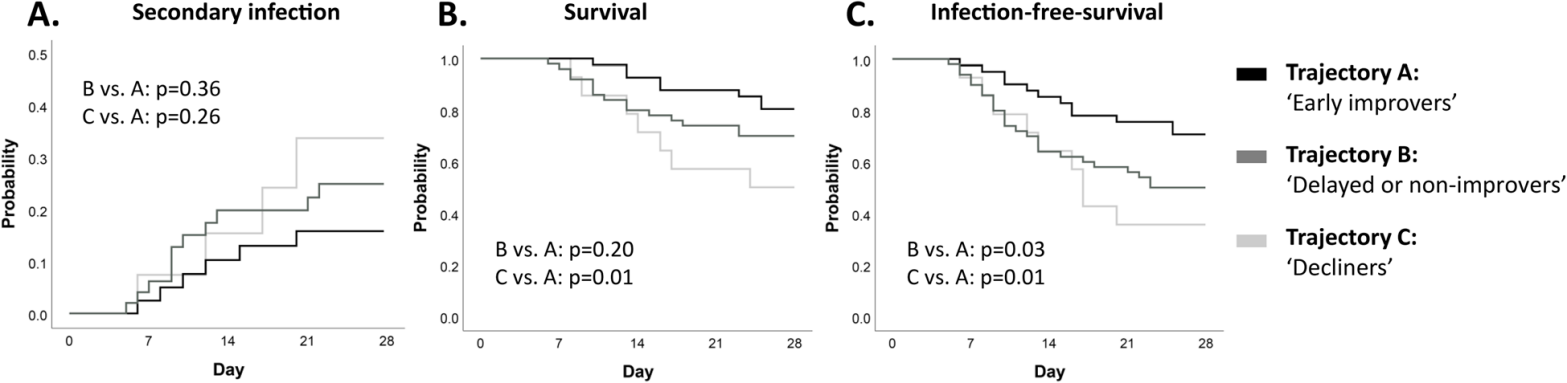
Hazard plot with a probability of **a** developing a secondary infections and Kaplan-Meier plot for **b** survival and **c** infection-free survival across the three mHLA-DR trajectories. *p* values were calculated using log-rank tests

Unsupervised cluster analysis produced four distinct mHLA-DR expression trajectories (individual data depicted in Supplementary Fig. 3). One of the identified trajectories contained only 4 patients, all with mHLA-DR values that appeared supraphysiological (> 30.000 AB/cell). Therefore, this trajectory was excluded from further analyses. The three final trajectories are depicted in Fig. 3 and patient characteristics are listed in Supplementary Table 4. Trajectory A is characterized by a moderately suppressed mHLA-DR expression at baseline and a swift increase over time (‘early improvers’), whereas in trajectory B (‘delayed or non-improvers’), baseline mHLA-DR expression is lower and recovery is delayed or not present. In contrast to the recovery of mHLA-DR expression in trajectories A and B, baseline mHLA-DR expression in trajectory C is relatively high but decreases over time (‘decliners’). Since the early improvers had the lowest incidence of secondary infections and lowest mortality (Supplementary Table 4), this trajectory was used as the reference category in further analyses. No significant differences in the development of secondary infections was observed between trajectories (Fig. 4a and Table 2). However, the decliners had a significantly lower survival compared to the early improvers, whereas this difference was not significantly different in the delayed or non-improvers vs. the early improvers (Fig. 4B and Table 2). The probability for adverse outcome (defined as either the occurrence of a secondary infection or death) during 28 days of follow-up was significantly higher in both the decliners and the delayed or non-improvers compared to the early improvers (Fig. 4c). Unadjusted Cox proportional hazard analysis revealed similar results (Table 2). When adjusting for baseline SOFA score, the hazard ratio remained significantly higher for the decliners vs. the early improvers (Table 2).

**Table 2:** Cox proportional hazard analysis for secondary infections, mortality and adverse outcome

		Hazard ratio [95% CI] (unadjusted)	*p* value	Hazard ratio [95% CI] adjusted for baseline SOFA score	*p* value
**Secondary infections**	Decliners vs. early improvers	2.0 [0.6–7.2]	0.27	2.5 [0.6–9.4]	0.19
	Delayed or non-improvers vs. early improvers	1.6 [0.6–4.3]	0.37	1.5 [0.5–4.0]	0.45
**Mortality**	Decliners vs. early improvers	3.3 [1.2–9.2]	0.02	2.5 [0.8–7.8]	0.10
	Delayed or non-improvers vs. early improvers	1.7 [0.7–4.1]	0.20	1.7 [0.7–4.0]	0.26
**Adverse outcome (secondary infection or death)**	Decliners vs. early improvers	2.9 [1.2–6.9]	0.02	2.8 [1.1–7.1]	0.03
	Delayed or non-improvers vs. early improvers	2.1 [1.0–4.1]	0.04	2.0 [1.0–4.0]	0.057

*CI* confidence interval

## Discussion

In the current study, we evaluated mHLA-expression kinetics in the largest group of septic shock patients so far, using a standardized flow cytometry-based assay. We established that differences in sites of infection or causative pathogens are not associated with differences in mHLA-DR expression kinetics. Most importantly, using unsupervised clustering, we identified three mHLA-DR trajectories (‘early improvers’, ‘delayed or non-improvers’ and ‘decliners’), and these trajectories corresponded with adverse outcome (secondary infection or death), which was significantly higher in both the decliners and delayed or non-improvers vs. the early improvers. Our findings illustrate that trajectories provide a better understanding of the relationship between mHLA-DR expression and adverse outcome and thereby have additional value over single measurements.

Although no statistically significant between-group differences in kinetics were found, patients with abdominal infections showed an increase in mHLA-DR expression over time, whereas this was not the case in the respiratory focus group. This is likely the result of the higher disease severity in patients with a respiratory infection, as we also demonstrate a significant correlation between SOFA score at admission and a decreased mHLA-DR expression at days 3–4 and 6–8. Of note, SOFA score was not associated with mHLA-DR expression at days 1–2, which suggests a delay in mHLA-DR downregulation. The association between disease severity and mHLA-DR expression is further supported by the fact that patients with a declining mHLA-DR expression (trajectory C) display a higher SOFA score, noradrenaline requirement and mortality rate compared to early improvers (trajectory A). In line with these results, others have also reported that disease severity is a main driver of sepsis-induced immunosuppression and sepsis outcome [14].

Previous work revealed that, in patients with bloodstream infections, those with *S. pneumonia* infections showed a swift increase in mHLA-DR expression over time, whereas patients with a *S. aureus* infection displayed a delayed recovery [11]. Patients with an *E. coli* infection had expression levels similar to those found in healthy volunteers [11]. However, the population was heterogeneous and the observed differences might therefore be explained by the large differences in disease severity between patients in the different pathogen categories [11]. In the current cohort of septic shock patients, we do not find differences in mHLA-DR expression kinetics between patients with Gram-positive, Gram-negative and negative cultures. This finding is strengthened by the fact that disease severity and outcome were similar across pathogen categories. A study using genome-wide gene expression analysis in mice revealed that Gram-positive and Gram-negative sepsis elicit common downstream pathways [15], which underscores that differences in mHLA-DR between these pathogen categories are not likely.

Overall, we demonstrate that patients with a decreasing mHLA-DR expression over time have the highest occurrence of secondary infections and mortality rate. This is in accordance with literature, as our group and others have previously described the relationship between decreased or non-recovering mHLA-DR expression and unfavourable outcome [6, 7, 16–21]. However, to the best of our knowledge, we are the first to apply unsupervised trajectory modelling. This data-driven ‘bottom-up’ approach might be more sensitive to identify endotypes of septic shock patients associated with unfavourable outcome. Previous results showing that recovery of mHLA-DR expression occurred less in non-survivors [16] are in line with our data showing that patients with decreasing mHLA-DR expression in trajectory C had a higher mortality. It is remarkable that, although the decliners had the highest disease severity at baseline, their mHLA-DR expression on days 1–2 was the highest. The absence of a correlation between mHLA-DR expression at days 1–2 and the severity of disease or any of the outcome parameters illustrates that a single measurement at this specific time point is of minor value. Furthermore, because the trajectories of the decliners and early improvers intersect at days 3–4, a single mHLA-DR measurement at days 3–4 is also not suitable to predict overall adverse outcome. These observations further indicate that it is the course of mHLA-DR over time (dynamic) rather than its value at a single time point (static), which is of clinical significance. To this end, patient enrichment in studies evaluating immunomodulatory treatments for sepsis-induced immunosuppression should be performed using dynamic mHLA-DR expression.

Strikingly, the overall mHLA-DR expression in our septic shock cohort was very low regarding the fact that the cut-off used for the treatment of sepsis-induced immunosuppression was 8000 AB/cell in a clinical trial [22]. In our cohort, 77% of the patients at days 3–4 and 68% at days 6–8 were below this threshold. These low values are in agreement with recent results obtained in clinical trials that included mHLA-DR monitoring [23, 24]. Thus, the identification of the appropriate threshold defining sepsis-induced immunosuppression requires further study, preferentially in a multicentre setting. It is likely that a cut-off for selecting the most severely affected patients will be more in the range of 5000 AB/cell than 8000 AB/cell. Despite the association with adverse outcome, the accuracy of low mHLA-DR expression to predict clinical outcome at the individual patient level was limited in our cohort. That said, considering the amount of literature associating low mHLA-DR and unfavourable outcomes, it remains a valuable marker for enrichment in clinical trials, for example to select a group of patients that may benefit from immunomodulating therapies [25, 26]. Nevertheless, in light of the fact that septic shock is a highly complex immunological syndrome, involving multiple immune defects that vary between patients and within patients over time [27], it remains debatable whether a single biomarker will ever be sufficient to gauge a patient’s overall immune status. Therefore, future research should be focused on the identification of combinations of biomarkers to classify immune endotypes in sepsis [2].

Strengths of the current study include the use of a large cohort comprised of only septic shock patients, which reduces variation compared to other studies in patients with multiple sepsis severities. Furthermore, we used a standardized mHLA-DR assay which facilitates generalizability and comparison with other cohorts using the same methodology. Finally, we used an innovative data-driven approach to identify mHLA-DR expression trajectories. Several limitations also need to be addressed. First, we performed the kinetics and trajectory analyses in subgroups of patients who remained in the ICU for at least 4 or 6 days, respectively. This introduced selection bias, as patients who died or were discharged at earlier time points were not taken into account. Second, disease severity parameters were only available at admission. A longer follow-up of these parameters would have allowed a more extensive analysis of the relationship between the kinetics of disease severity and mHLA-DR expression. Third, the number of patients with specific pathogens (e.g. *S. aureus* or *P. aeruginosa*), other groups of pathogens (e.g. fungi, viruses) and sites of infection other than abdominal, respiratory and urinary tract were too low to draw conclusions about mHLA-DR expression kinetics. Fourth, despite the large cohort of septic shock patients, the sample size of studied subgroups was relatively low, which might explain the lack of significance for secondary infections across the trajectories. Due to small sample sizes in subgroups, the absence of a statistical difference cannot rule out the presence of an association. Finally, approximately 60% of the patients in our cohort underwent a surgical intervention, which can impact mHLA-DR expression [28].

## Conclusions

The site of primary infection and different groups of pathogens are not associated with different mHLA-DR expression kinetics in septic shock patients. We identified three distinct mHLA-DR expression trajectories, which were related to clinical outcome. The risk for adverse outcome (secondary infection or death) was significantly higher in patients with overall declining or delayed or non-improving mHLA-DR expression compared to patients with a swift increase. Our study signifies that changes in mHLA-DR expression over time and not absolute values or static measurements are of clinical importance in septic shock patients.

## Supplementary information

**Additional file 1.** Leijte_Revised Supplementary Material.docx. Supplementary Methods (exclusion criteria, data collection, analysis of HLA-DR expression, unsupervised trajectory analysis). Supplementary Tables. Supplementary Figures.

## Data Availability

The datasets used and/or analysed during the current study are available from the corresponding author on reasonable request.

## Abbreviations

(m)HLA-DR: Monocytic Human Leukocyte Antigen – DR isotype
ICU: Intensive care unit
ANOVA: Analysis of variance
SOFA: Sequential Organ Failure Assessment
SAPS: Simplified Acute Physiology Score
AB/c: Antibodies per cell
AUROC: Area under the receiver operating characteristics
CI: Confidence interval
(a)HR: Adjusted hazard ratio
