# Supplementary material for: Monocytic HLA-DR expression kinetics in septic shock patients with different pathogens, sites of infection and adverse outcomes
Source: Crit Care. 2020 Mar 20;24:110. doi: 10.1186/s13054-020-2830-x (PMC7082984; doi:10.1186/s13054-020-2830-x)
Supplement: Supplementary file 1 — Additional file 1. Leijte_Revised Supplementary Material.docx. Supplementary Methods (exclusion criteria, data collection, analysis of HLA-DR expression, unsupervised trajectory analysis). Supplementary Tables. Supplementary Figures. [file 13054_2020_2830_MOESM1_ESM.docx]

**Supplementary Material**

**Title manuscript:** Monocytic HLA-DR expression kinetics in septic shock patients with different pathogens, sites of infection and adverse outcomes

**Authors**

Guus P. Leijte^1,2^, Thomas Rimmelé^3,4^, Matthijs Kox^1,2^, Niklas Bruse^1,2^, Céline Monard^4^, Morgane Gossez^3,5^, Guillaume Monneret^3,5^, Peter Pickkers^1,2^, Fabienne Venet^3,5*^

**Affiliations**

^1^Dept. of Intensive Care Medicine, Radboud university medical center, Nijmegen, The Netherlands.

^2^Radboud Center for Infectious Diseases, Radboud university medical center, Nijmegen, The Netherlands.

^3^Pathophysiology of Injury-Induced Immunosuppression, Université Claude Bernard Lyon 1, Hospices Civils de Lyon, bioMérieux, Edouard Herriot Hospital, Lyon, France.

^4^Hospices Civils de Lyon, Edouard Herriot Hospital, Anesthesia and Critical Care Medicine Department, Lyon, France.

^5^Hospices Civils de Lyon, Edouard Herriot Hospital, Immunology Laboratory, Lyon, France.

**Corresponding author*

**Content of Supplementary Material**

**Supplementary Methods**

- Exclusion criteria Page 3
- Data collection Page 3
- Analysis of HLA-DR expression on monocytes Page 4
- Unsupervised trajectory analysis Page 4

**Supplementary Tables:**

- Supplementary Table 1 Page 6
- Supplementary Table 2 Page 7
- Supplementary Table 3 Page 8
- Supplementary Table 4 Page 9
- Supplementary Table 5 Page 11

**Supplementary Figures**

- Supplementary Figure 1 Page 12
- Supplementary Figure 2 Page 13
- Supplementary Figure 3 Page 16
- Supplementary Figure 4 Page 17
- Supplementary Figure 5 Page 17
- Supplementary Figure 6 Page 18

**References** Page 19

**Supplementary Methods**

*Exclusion criteria*

Exclusion criteria were presence of a pre-existent condition or treatment that could influence the immune status (e.g. HIV infection, use of immunosuppressive medication), hematological disease or a solid tumor within 5 years prior inclusion, extracorporeal circulation, pregnancy, aplasia, institutionalized patients and patients under the age of 18 years. Patients were not included when their day 3-4 sample was scheduled during the weekend.

*Data collection*

ICU admission data (e.g. demographic characteristics, comorbidities, SOFA score, SAPS II score, site of infection, pathogens) and follow-up data regarding the need for supportive interventions (e.g. mechanical ventilation) or medication (e.g. noradrenaline) were collected. During their ICU stay, patients were screened daily for exposure to invasive devices (intubation, indwelling urinary catheter and central venous line). A secondary (nosocomial) infection was defined as an ICU-acquired infection that developed after patient inclusion. Secondary infections were microbiologically-proved pneumonia, urinary tract infection, bacteraemia and infection of an intravascular device. All cases were reviewed and confirmed by an intensivist who was blinded to the mHLA-DR results, using the definitions proposed by the committee for nosocomial infections and healthcare associated infections (CNTILS) of the French Ministry of Health. Survival status at discharge from the ICU or hospital, and at 28 days following inclusion was recorded. The local ethical board (#IRB11236) waived the need for written informed consent for this observational study. However, oral information and non-opposition to inclusion in the study were mandatory and were systematically obtained before any study procedure was performed.

*Analysis of HLA-DR expression on monocytes*

Ethylenediaminetetraacetic acid (EDTA-)anticoagulated blood was drawn at day 1-2, day 3-4, and day 6-8 following study inclusion. Blood was stored at 4-8 ^o^C and processed within 2 hours after withdrawal. The expression of mHLA-DR was determined using the Anti-HLA-DR/Anti-Monocyte Quantibrite assay (BD Biosciences, San Jose, USA) on a Navios flow cytometer (Beckman Coulter, Hialeah, FL) and flow data were analysed using Navios software (Beckman Coulter). Total number of antibodies bound per monocyte (AB/cell) were quantified using calibration with a standard curve determined with BD Quantibrite phycoerythrin (PE) beads (BD Biosciences) as described elsewhere (1).

*Unsupervised trajectory analysis*

An R package (*crimCV*) was used to fit finite mixtures of Zero-Inflated Poisson (ZIP) regression models on longitudinal data in order to calculate group-based trajectories. The *crimCV* package allows setting the number of polynomials and the number of trajectories. To choose the best model and evaluate the performance of the chosen model, we used three model selection criteria: the Akaike information criterion (AIC), the Bayesian information criterion (BIC) and the cross-validation error (CV) with Jackknife resampling. The amount of polynomials was set at 2. Thereafter, we created 100 bootstrap samples (with replacement) with the amount of clusters set at 1, 2, 3, 4 and 5. The median [95%-CI] of the AIC, BIC and CV of the models are displayed in Supplementary Figure 4. The distribution of patients to each of the trajectories for the models is displayed in Supplementary Figure 5. The Kaplan Meier plot for the 3 and 5-trajectory model is depicted in Supplementary Figure 6. The models using 3, 4 and 5 clusters show great overlap in clustering and clinical outcome. However, the 3-cluster model still has considerable variation within the clusters, whereas the 5-cluster model ‘overclusters’ our data and thereby limits the translation to the research/clinical setting. To this end, the 4-cluster model used for further analysis.

**Supplementary Tables**

**Supplementary Table 1. Site of primary infection in the cohort of 241 septic shock patients**

| **Site of infection** | **Number (% of total cohort)** |
| --- | --- |
| Abdominal | 107 (44.4) |
| Respiratory | 44 (18.3) |
| Urinary Tract | 29 (12.0) |
| Soft tissue | 19 (7.9) |
| Deep wound infection | 11 (4.6) |
| Oropharyngeal | 7 (2.9) |
| Bacteraemia (catheter-related) | 6 (2.5) |
| Joints | 4 (1.7) |
| Other/unspecified | 3 (1.2) |
| Thorax | 2 (0.8) |
| Central nervous system | 2 (0.8) |
| Unknown | 7 (2.9) |
| **Total** | 241 (100) |

**Supplementary Table 2. Pathogens cultured in the cohort of 241 septic shock patients.**

| **Pathogen** | **Number (% of total cohort)** |
| --- | --- |
| Gram-positive | 64 (27) |
| Gram-negative | 77 (32) |
| Fungi | 6 (2) |
| Virus | 5 (2) |
| Multiple categories | 24 (10) |
| Culture negatives | 65 (27) |
| **Total** | 241 (100) |

**Supplementary Table 3. Location and incidence of secondary infections in the cohort of 241 septic shock patients.**

| **Site of secondary infection** | **Number (% of total cohort)** |
| --- | --- |
| Respiratory | 19 (60) |
| Urinary tract | 3 (9) |
| Bacteraemia | 4 (13) |
| Catheter-related | 3 (9) |
| Unknown | 3 (9) |
| **Total** | 32 (13) |

**Supplementary Table 4. Characteristics of septic shock patients within each trajectory.**

|  | **Trajectory A:**  **‘Early improvers’**  **(n=41)** | **Trajectory B**  **‘Delayed or non-improvers’**  **(n=50)** | **Trajectory C**  **‘Decliners’**  **(n=14)** | **p-value** |
| --- | --- | --- | --- | --- |
| **Baseline** |  |  |  |  |
| Gender (male) | 27 (66%) | 34 (68%) | 7 (50%) | 0.45 |
| Age (years) | 66 [60-76] | 69 [63-80] | 73 [67-80] | 0.14 |
| BMI | 26 [22-29] | 26 [24-31] | 24 [21-29] | 0.36 |
| SOFA score (<24hr) | 9 [9-11] | 10 [8-13] | 12 [8-14] | 0.047 |
| SAPS II score (<24hr) | 63 [53-71] | 64 [55-77] | 63 [52-79] | 0.32 |
| Lactate (mmol/L) | 3.4 [2.5-4.8] | 4 [2.7-6] | 4.3 [2.6-7.2] | 0.08 |
| **Interventions** |  |  |  |  |
| Noradrenaline (µg/kg/min)^#^ | 0.8 [0.4-1.4] | 0.8 [0.4-1.4] | 1.2 [0.8-2.9] | 0.03 |
| Hydrocortisone | 21 (51%) | 31 (62%) | 9 (64%) | 0.51 |
| Mechanical ventilation | 36 (88%) | 46 (92%) | 11 (79%) | 0.37 |
| Length of MV (days) | 4 [2-12] | 7 [3-15] | 8 [0-15] | 0.26 |
| Renal replacement therapy | 11 (27%) | 13 (26%) | 3 (21%) | 0.92 |
| Post-surgery admission | 31 (76%) | 38 (76%) | 4 (29%) | 0.002 |
| **Outcome measures** |  |  |  |  |
| Secondary infection | 6 (15%) | 14 (28%) | 4 (29%) | 0.28 |
| Length of ICU stay (days) | 13 [7-22] | 14 [10-24] | 14 [9-18] | 0.25 |
| Length of hospital stay (days) | 31 [21-52] | 27 [15-45] | 20 [11-44] | 0.33 |
| 28-day mortality | 8 (19%) | 15 (30%) | 7 (50%) | 0.09 |
| ICU mortality | 5 (12%) | 14 (28%) | 8 (57%) | 0.004 |
| Hospital mortality | 6 (15%) | 17 (34%) | 8 (57%) | 0.007 |
| Infection-free-28-day-survival^†^ | 29 (71%) | 25 (50%) | 5 (36%) | 0.04 |
| **Site of infection** |  |  |  |  |
| Respiratory | 6 (15%) | 6 (12%) | 4 (29%) | 0.29 |
| Abdominal | 18 (44%) | 24 (48%) | 3 (21%) |  |
| Urinary tract | 2 (5%) | 1 (2%) | 1 (7%) |  |
| **Pathogen category** |  |  |  |  |
| Gram-positive | 9 (22%) | 9 (18%) | 2 (14%) | 0.98^1^ |
| Gram-negative | 12 (29%) | 18 (36%) | 3 (22%) | 0.76^2^ |
| Fungal | 2 (5%) | 3 (6%) | 1 (7%) |  |
| Virus | 1 (2%) | 1 (2%) | 1 (7%) |  |
| Multiple categories | 7 (17%) | 7 (14%) | 2 (14%) |  |
| Culture negatives | 10 (24%) | 12 (24%) | 5 (36%) |  |

Data are presented as frequencies and percentages (%) for categorical data and medians and interquartile ranges [IQR] for continuous variables. P-values were calculated using Chi-Square tests for categorical data and one-way ANOVA on log-transformed continuous data. ^1^Chi-Square test for Gram-positive, Gram-negative and negative cultures only. ^2^Chi-Square test for all six pathogen categories listed. ^#^Maximum dose within the first 24 hours after study inclusion. ^†^Alive at day 28 without development of secondary infection. BMI = body mass index; SOFA = sequential organ failure assessment; SAPS = simplified acute physiology score; MV = mechanical ventilation; ICU = intensive care unit.

**Supplementary Table 5. Specification of imputed values for the subgroups.**

|  |  |  | **Reason for imputation** | | |
| --- | --- | --- | --- | --- | --- |
|  | **Group size** | **Total imputations**  (% within group) | **Patient**  **died** | **Patient discharged** | **Other^#^** |
| **Total cohort** | 167 | 59 (35%) | 16 (27%) | 37 (63%) | 6 (10%) |
| **Site of infection** |  |  |  |  |  |
| Respiratory group | 24 | 9 (38%) | 5 (56%) | 2 (22%) | 2 (22%) |
| Abdominal group | 77 | 21 (27%) | 3 (14%) | 16 (76%) | 2 (10%) |
| Urinary tract group | 18 | 12 (67%) | 2 (17%) | 10 (83%) | 0 (0%) |
| **Pathogen category** |  |  |  |  |  |
| Gram-positive | 42 | 21 (50%) | 5 (24%) | 12 (57%) | 4 (19%) |
| Gram-negative | 55 | 20 (36%) | 6 (30%) | 12 (60%) | 2 (10%) |
| Culture negative | 41 | 14 (34%) | 4 (29%) | 10 (71%) | 0 (0%) |

^#^Laboratory closed/absence of personnel, assay failure.

**Supplementary Figures**


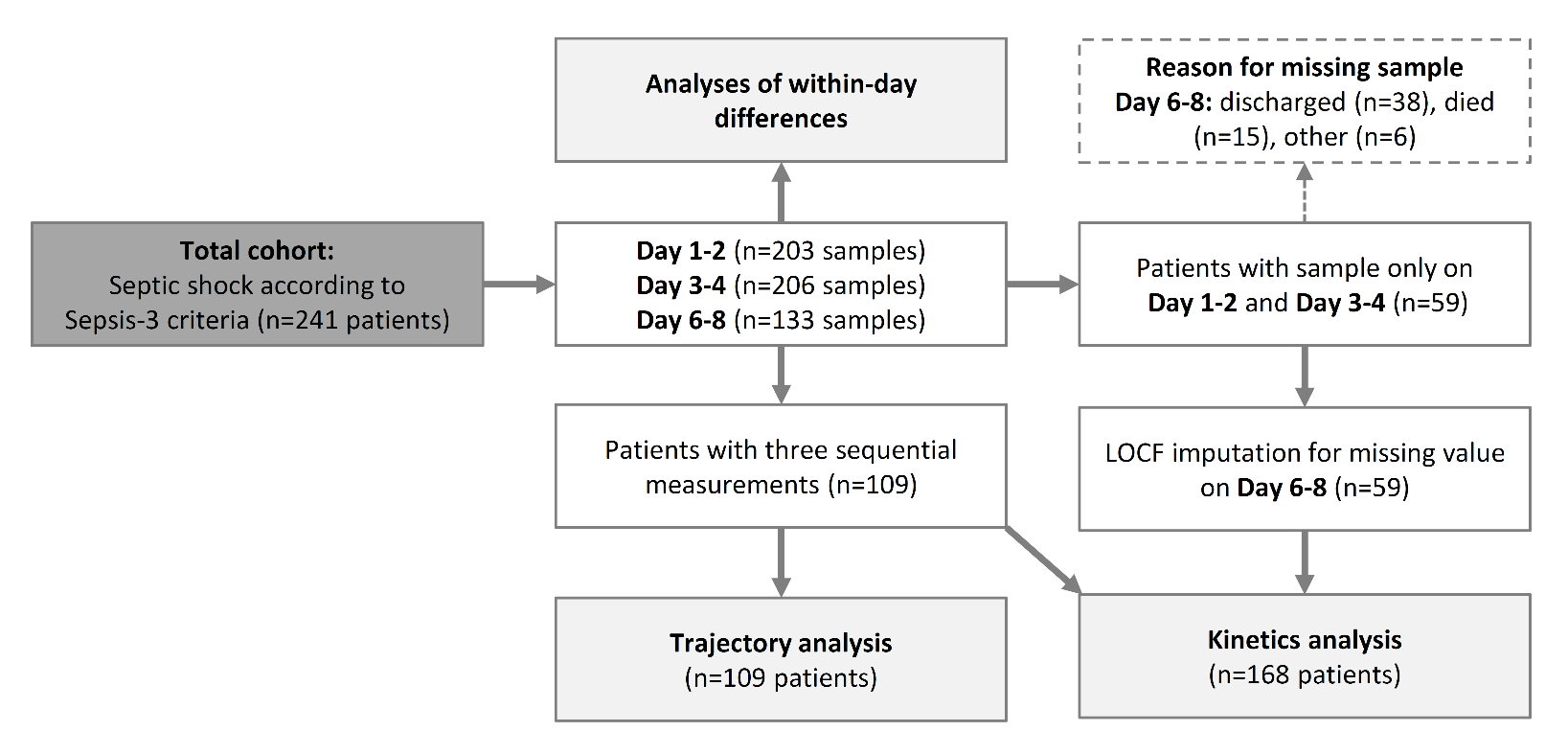


**Supplementary Figure 1.** Flowchart of the patient datasets used for the different analyses.


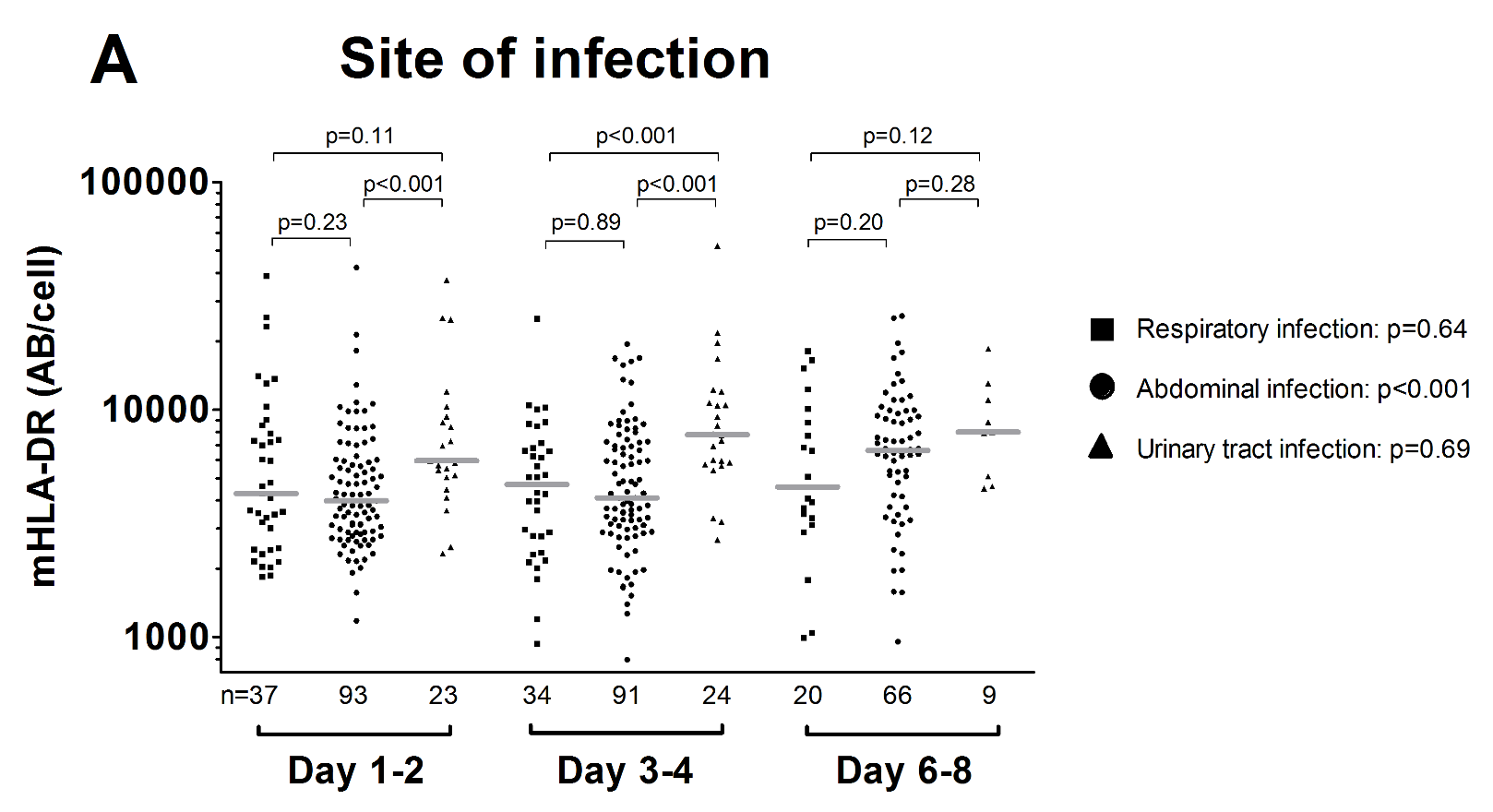


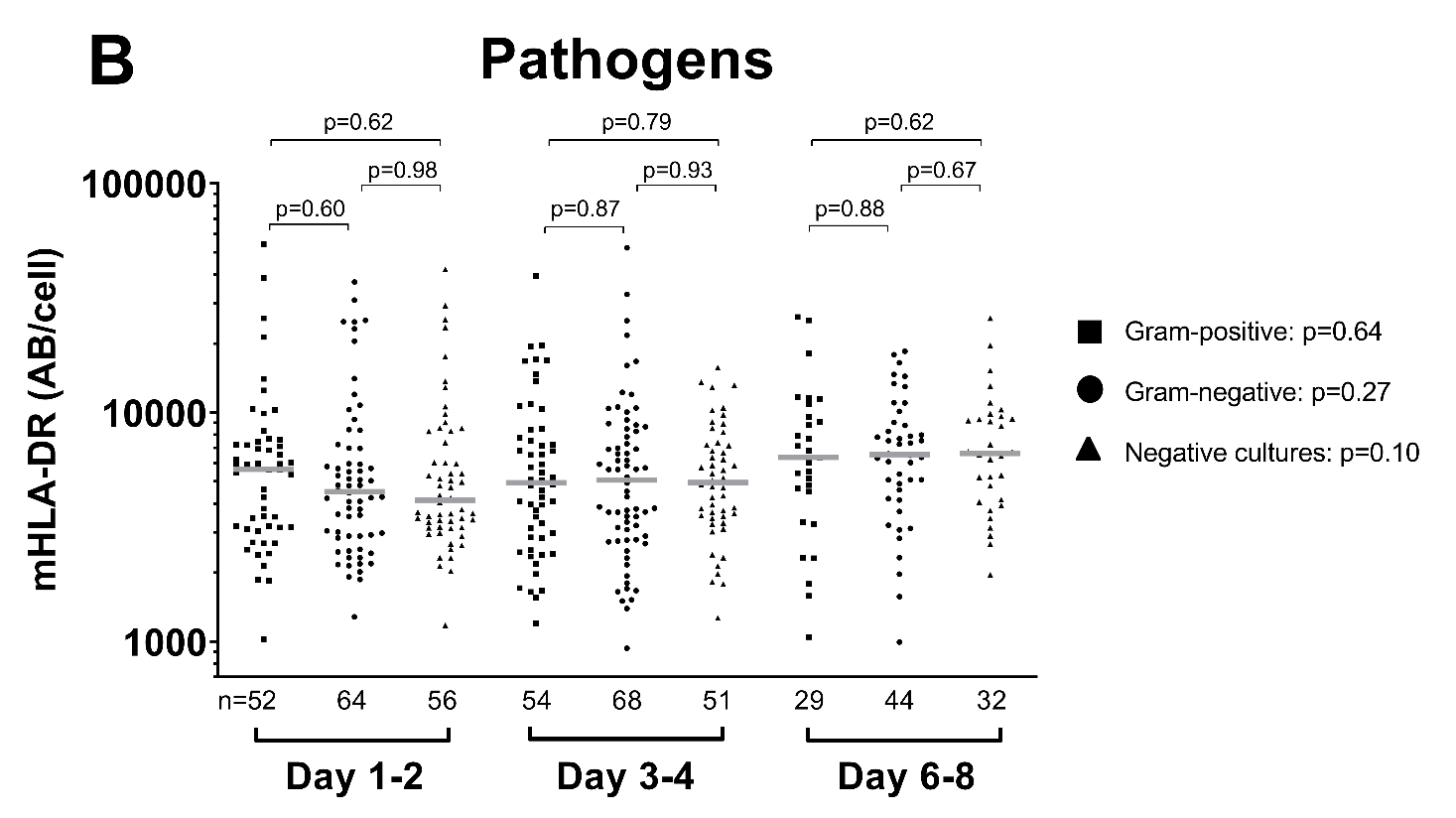


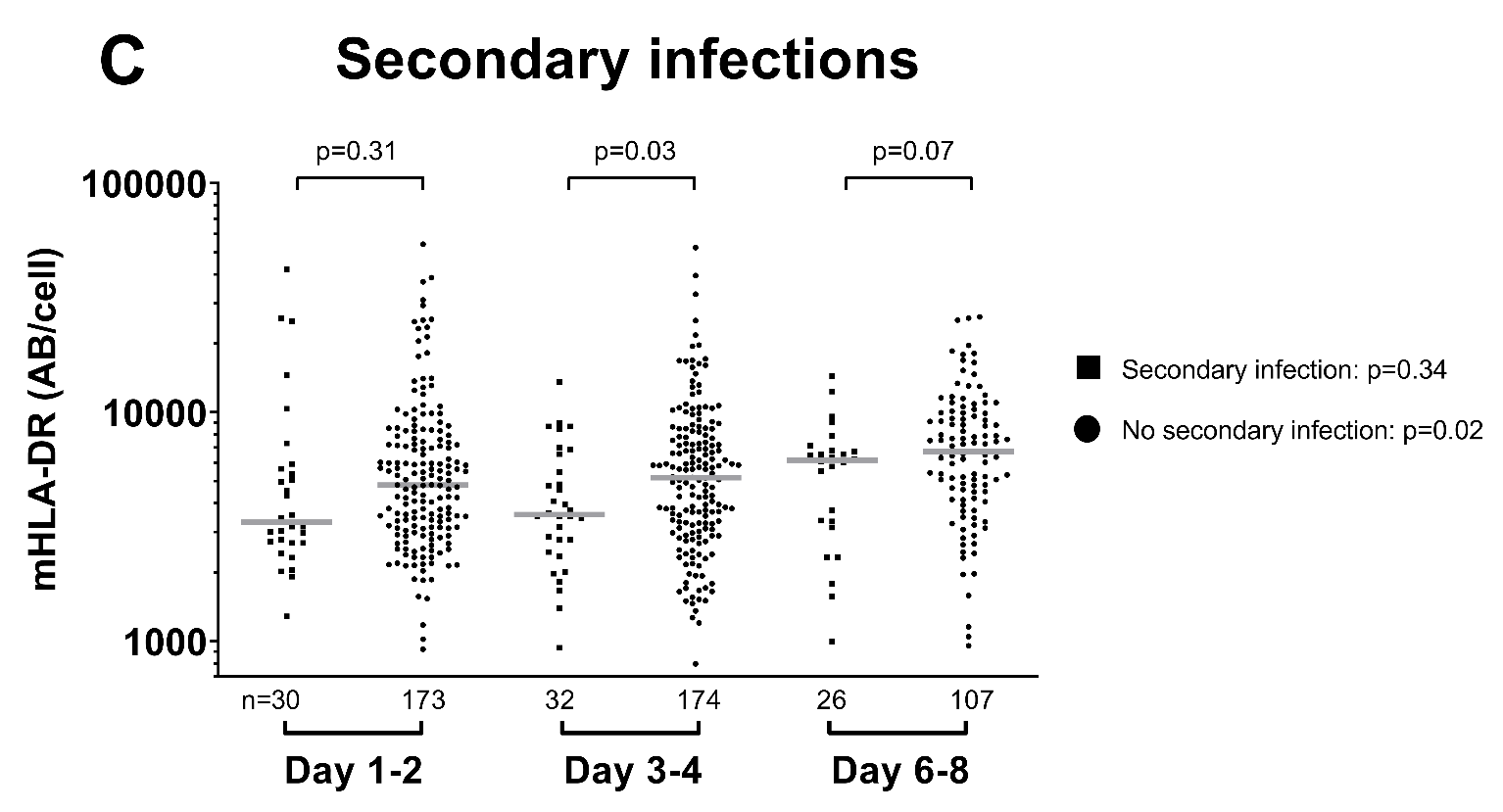


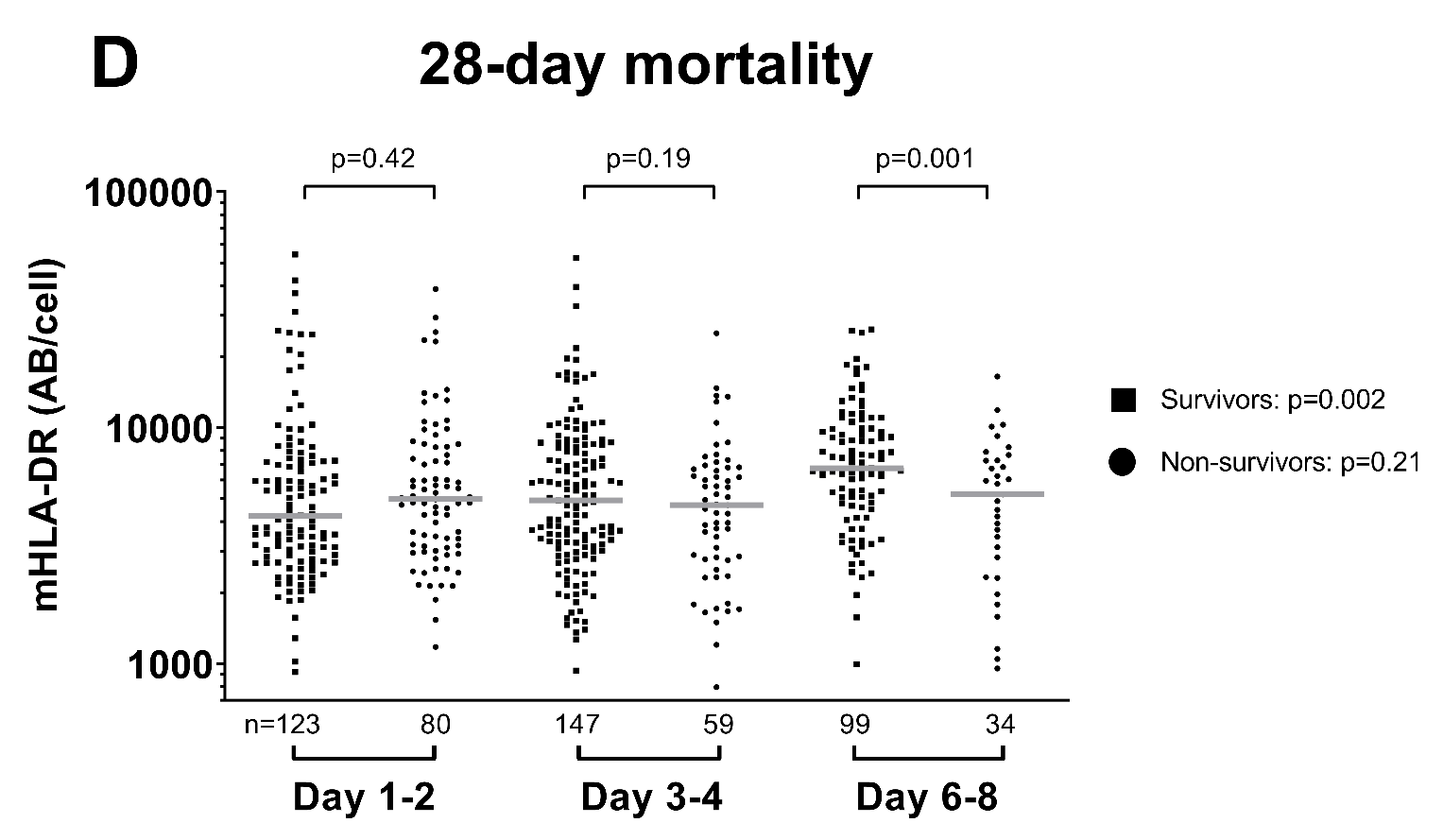


**Supplementary Figure 2.** Scatter plots of mHLA-DR expression on day 1-2, day 3-4, and day 6-8 for patients with (**A**) respiratory, abdominal and urinary tract infections, (**B**) Gram-positive and Gram-negative bacteria, and negative cultures (**C**) secondary infections versus no secondary infection, and (**D**) 28-day survivors versus non-survivors. P-values next to group designation represent within-group differences over time were calculated using one-way ANOVA on log-transformed data. P-values between groups within each timepoint were calculated using Student’s T test on log-transformed data. The horizontal grey line represents the median.


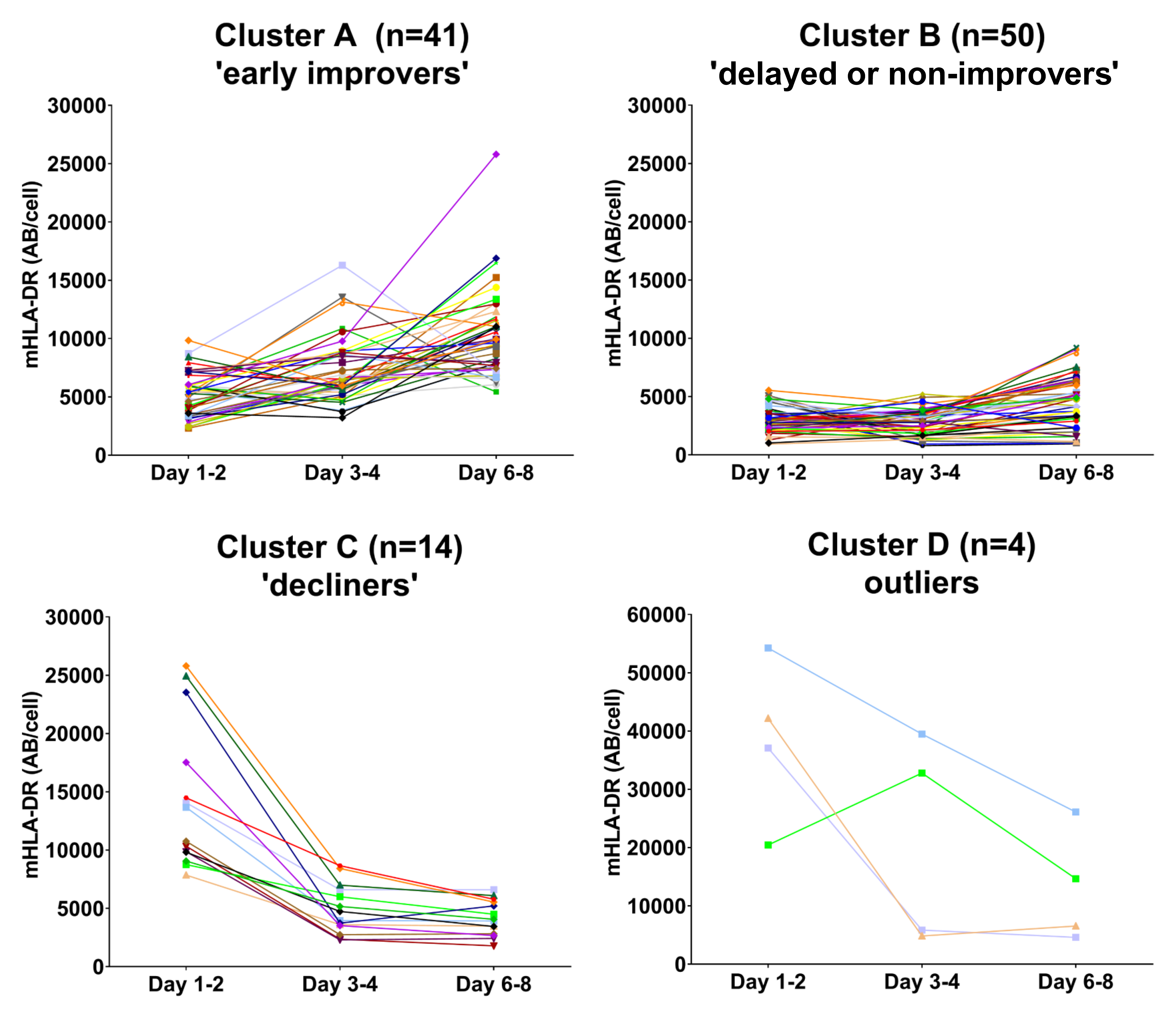


**Supplementary Figure 3.** mHLA-DR kinetics of individuals (n=109) allocated to one of the four trajectories.


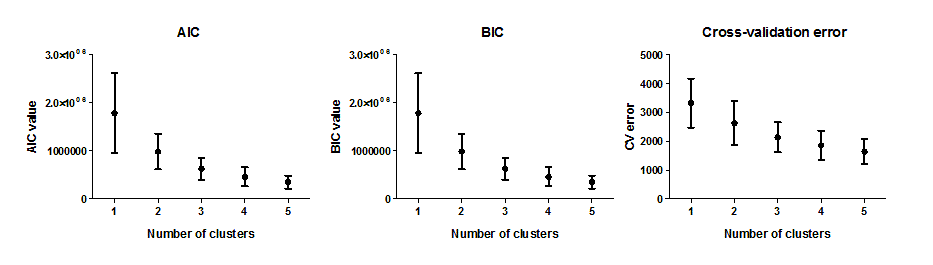


**Supplementary Figure 4.** The model selection criteria AIC, BIC and CV for the model with 1, 2, 3, 4, and 5 trajectories.


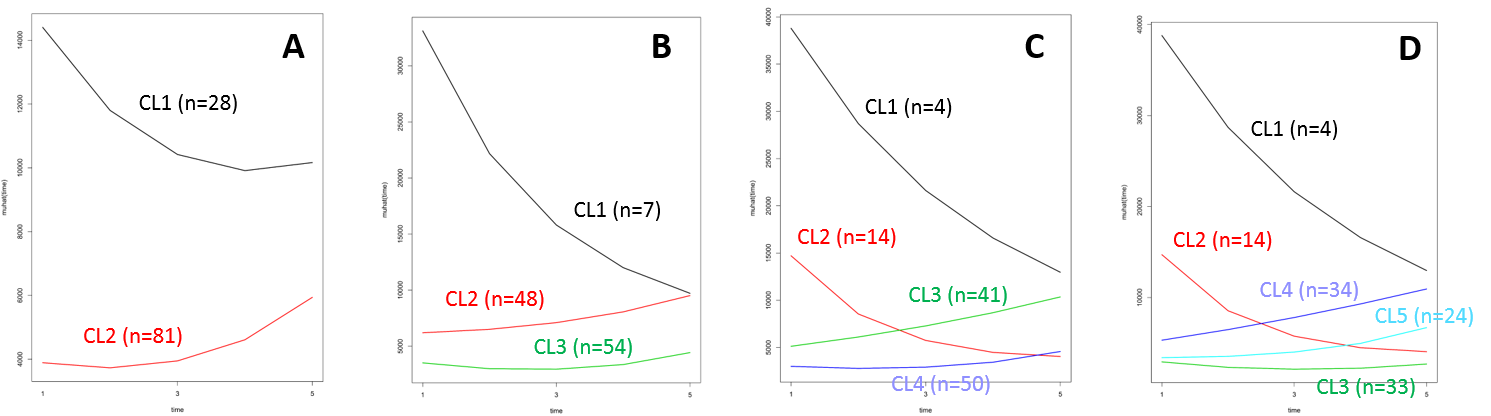


**Supplementary Figure 5.** Distribution of patients within model with (**A**) 2, (**B**) 3, (**C**) 4, and (**D**) 5 trajectories.


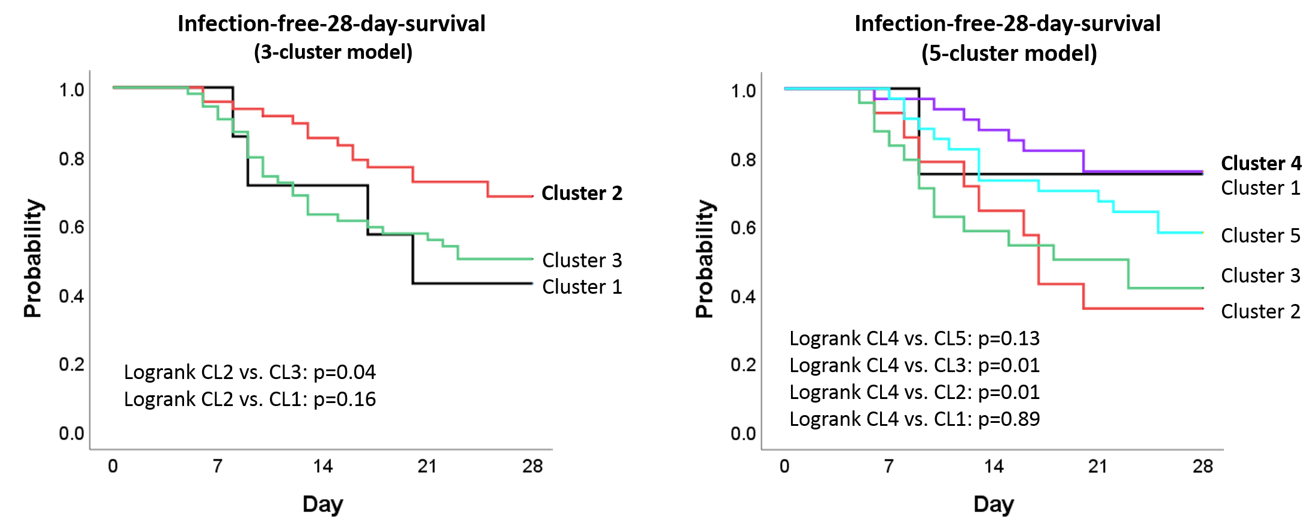


**Supplementary Figure 6.** Kaplan Meier plot for adverse outcome (infection or death) for each of the trajectories within the (**A**) 3-trajectory model and (**B**) 5-trajectory model. Cluster 2 (3-cluster model) and cluster 4 (5-trajectory model) were used as reference category (early improvers).

**References**

1. Demaret J, Walencik A, Jacob MC, Timsit JF, Venet F, Lepape A, et al. Inter-laboratory assessment of flow cytometric monocyte HLA-DR expression in clinical samples. Cytometry Part B, Clinical cytometry. 2013;84(1):59-62.
